# Hydrogen Sulfide Plays an Important Protective Role through Influencing Endoplasmic Reticulum Stress in Diseases

**DOI:** 10.7150/ijbs.38143

**Published:** 2020-01-01

**Authors:** Honggang Wang, Xingzhuo Shi, Mengyuan Qiu, Shuangyu Lv, Huiyang Liu

**Affiliations:** 1 Institute of Biomedical Informatics, Bioinformatics Center, School of Basic Medical Sciences, Henan University, Kaifeng, Henan, 475000, China.; 2 School of Life Science, Henan University, Kaifeng, Henan, 475000, China.

**Keywords:** Hydrogen sulfide, endoplasmic reticulum stress, cardiomyopathy, neurological diseases, respiratory diseases, vascular diseases

## Abstract

The endoplasmic reticulum is an important organelle responsible for protein synthesis, modification, folding, assembly and transport of new peptide chains. When the endoplasmic reticulum protein folding ability is impaired, the unfolded or misfolded proteins accumulate to lead to endoplasmic reticulum stress. Hydrogen sulfide is an important signaling molecule that regulates many physiological and pathological processes. Recent studies indicate that H_2_S plays an important protective role in many diseases through influencing endoplasmic reticulum stress, but its mechanism is not fully understood. This article reviewed the progress about the effect of H_2_S on endoplasmic reticulum stress and its mechanisms involved in diseases in recent years to provide theoretical basis for in-depth study.

## 1. Introduction

**Figure 1:**
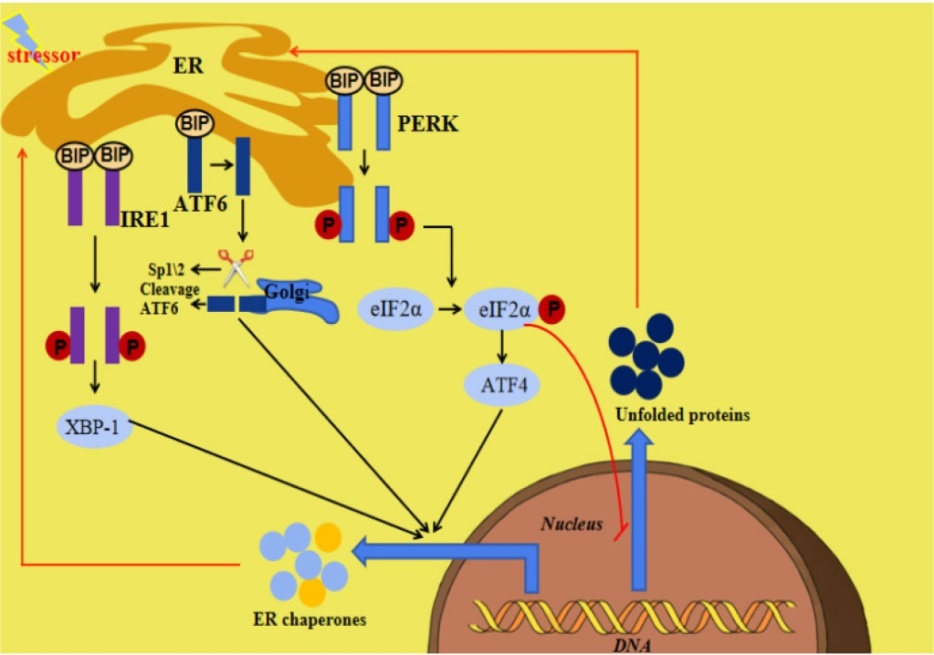
** Summary of ERS and the UPR.** When ERS is activated, there are three parallel signaling branches in UPR. ATF4, activating transcription factor 4; ATF6, activating transcription factor 6; BIP, binding immunoglobulin protein; ER, endoplasmic reticulum; ERS, endoplasmic reticulum stress; IRE1,inositol-requiring protein 1; PERK, PRKR-like ER kinase; SP1, site-1 protease; UPR, unfolded protein response; XBP1, X-box binding protein 1.

The endoplasmic reticulum (ER) is an important organelle responsible for protein synthesis, modification, folding, assembly and transport of new peptide chains 1-5. In addition, it regulates the cholesterol and lipid-membrane biosynthesis and the signaling mechanisms of cell surviving and death 6, 7. Under stress conditions including glucose deficiency, environmental toxins, viral infection, changes in Ca^2+^ levels, hypoxia, inflammation and oxidative stress, ER homeostasis can be interrupted, which is termed ER stress (ERS). ERS is defined as the disturbance of ER function, which interferes with protein folding, post-translational modification and secretion. Finally, the accumulation of unfolded proteins in ER initiates a homeostatic signaling network called as the unfolded proteins reaction (UPR) 8, 9. When the perturbation is moderate, UPR activation will promote a homeostatic recovery of ER and help cells adapt to changes. However, if the interference is intense and prolonged, ERS and UPR will initiate the death signaling pathway, which will lead to the onset of various diseases 10. The ERS and UPR are mediated by three transmembrane ER signaling proteins: pancreatic endoplasmic reticulum kinase (PERK), inositol-requiring enzyme 1 (IRE1) and activating transcription factor 6 (ATF6), which mediate three parallel signal branches respectively 11, 12. Under non-pressure conditions, the binding immunoglobulin (BIP) binds to PERK, IRE1 and ATF6 to stabilize and prevent their activation. The stressors and unfolded proteins promote the isolation of BIP from PERK, IRE1 and ATF6, thereby activating these three molecules. Subsequently, the autophosphorylated PERK phosphorylates eIF2a to inhibit mRNA translation and global protein synthesis, and increase ATF4 expression, the activated IRE1 cleaves Xbp1 mRNA and the isolated ATF6 is cleaved by 1-site protease (sp1) and 2-site protease (sp2) proteins in Golgi complex. At last, the cleaved Xbp1, the ATF4 and the spliced ATF6 promote the expression of ER chaperone genes, which are further involved in eliminating unfolded proteins and restoring homeostasis in normal cells (Figure 1) 10. Many diseases have been reported to be related with ERS 13, 14.

**Figure 2:**
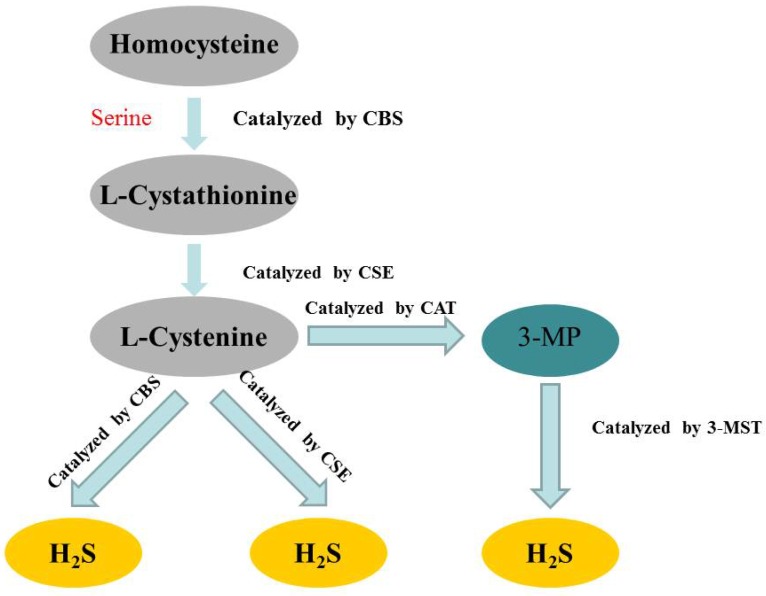
** Summary of the production process of endogenous H_2_S.** CBS: cystathionine-beta-synthase; CSE: cystathionine-gamma-lyase; 3-MST: 3-mercaptopyruvate thiotransferase; 3-MP: 3-mercaptopyruvate; CAT: cysteine aminotransferase.

Hydrogen sulfide (H_2_S) has long been considered as a flammable, water-soluble, colorless and toxic gas. However, since the 1990s, more and more studies have confirmed that H_2_S belonged to a class of gasotransmitters, together with nitric oxide (NO) and carbon monoxide (CO) 15-17. In mammalian cells, H_2_S is produced by endogenous enzymatic and non-enzymatic pathways. The enzymatic generation of H_2_S, which may be important for the regulation in given cells under special conditions, is the focus of the research. Several different mammalian enzymatic systems for H_2_S production have been described in detail. Most commonly, three typical H_2_S-producing enzymes are identified: cystathionine-gamma-lyase (CSE), cystathionine-beta-synthase (CBS) and 3-mercaptopyruvate thiotransferase (3-MST) 18-20. Cystathionine is produced by β-substitution reaction of homocysteine with serine catalyzed by CBS. CSE catalyzes the elimination of α, γ-cysteine of cystathionine to produce cystenine. Under the catalysis of CBS and CSE, cysteine can form H_2_S through β elimination reaction. 3-mercaptopyruvate (3-MP) is produced by transferring amines from cystine to α-ketoglutarate via cysteine aminotransferase (CAT). 3-MST catalyzes the sulphur of 3-MP to convert into H_2_S 21 (Figure 2). The biological function of H_2_S does not depend on H_2_S itself, but on the formation of new molecules, such as S-nitrosothiols, whose possible mechanisms include reversible protein sulfidation 22. H_2_S has many physiological functions, such as relaxing blood vessels, lowering blood pressure 23, 24, anti-apoptotic 25, anti-inflammatory 26, anti-oxidative stress and regulation of ER stress 27. The role of H_2_S in the regulation of ERS has been one of the focuses of attention in recent years 28.

In this review, we summarize the progress about the effects of H_2_S on ERS and the mechanism involved in recent years to provide ideas for relevant basic research in the future.

## 2. H_2_S plays cardioprotective role by influencing endoplasmic reticulum stress

Diabetic cardiomyopathy (DCM) is one of the major cardiac complications independent of coronary artery disease and hypertension in diabetic patients 29. In recent years, many studies have shown that ERS plays a crucial role in the occurrence and development of DCM 30, 31. Hyperglycemia induces cardiomyocyte apoptosis by activating ERS through caspase-12 dependent pathway and C/EBP-homologous protein (CHOP) dependent pathway 32-34. Fang Li and her coworkers reported that in streptozotocin (STZ)-induced diabetic rats, ERS was increased by hyperglycemia, leading to myocardial fibrosis and cardiomyocyte apoptosis. While treatment with H_2_S reduced ERS to inhibit myocardial apoptosis and improve myocardial fibrosis, suggesting that H_2_S had a potential role in the treatment of DCM 35. In this experiment, since the intervention of H_2_S is simultaneous with the establishment of DC model, not after the establishment of DC model, thus, the protective effect of H_2_S on DC cannot be fully demonstrated. Whether H_2_S regulated ERS to play a protective effect on DC remained to be studied. Moreover, long-term hyperglycemia can cause excessive production of reactive oxygen species (ROS) in mitochondria of cardiomyocyte 36, 37 and excessive ROS induces ERS which leads to cardiomyocyte apoptosis 38. H_2_S can inhibit the production of ROS, indicating that H_2_S can regulate ERS through ROS. ER and mitochondria are spatially close organelles which are joined together by ER-mitochondrial associated membranes 39. ROS regulate ER-mitochondrial crosstalk during ERS-induced apoptosis 40. In streptozotocin (STZ)-induced diabetic rats, H_2_S reduces ROS in mitochondria and ERS-induced myocardial apoptosis through regulating ER-mitochondrial crosstalk 41. ROS associates the antioxidant effect of H_2_S with its inhibition of ERS. Researches showed that the excessive lipid deposition and ERS might play a role in the pathogenesis of DCM 42-44. In the hearts of STZ-induced rats or in AC16 cardiac cells treated with palmitic acid (PA), endogenous H_2_S decreased, ERS, apoptosis and lipid accumulation increased, suggesting that endogenous H_2_S, ERS and lipotoxicity are involved in the pathological process of DCM. The further experiment showed that exogenous H_2_S alleviated myocardial lipotoxicity and ER stress. The similar results can be obtained by using ERS inhibitors (4-PBA), suggesting that exogenous H_2_S inhibits lipid accumulation and myocardial toxicity through suppressing ERS 45. This is consistent with previous reports that H_2_S mitigated high fat diet-induced cardiac dysfunction through suppression of ERS 46. The mechanism of the effect of ERS on myocardial lipotoxicity of DCM remains to be studied. Myocardial ischemia reperfusion (I/R) injury is an important cause of myocardial injury 47. Recently, it has been proved that ERS is related to myocardial I/R injury 48. Myocardial I/R decreased endogenous H_2_S, increased ERS and ERS-induced cardiomyocyte apoptosis. H_2_S preconditioning could reverse these above changes. Moreover, pretreatment with ERS inhibitors yielded similar results as H_2_S. Collectively, these results indicated that H_2_S ameliorated myocardial I/R injury by attenuating excessive ERS induced by myocardial I/R 49.This added a new mechanism, which remains to be studied, to the myocardial protection of H_2_S. Several studies suggest that chronic intermittent hypoxia (CIH) may induce ER stress and lead to myocardial injury 50-52. Zhou, et al. reported that CIH induced myocardial injury by activating ERS, while the treatment with the inhibitor of cystathionine γ-lyase (DL-propargylglycine, PAG) alleviated myocardial injury induced by CIH 53. This result is inconsistent with previous study that H_2_S could alleviate myocardial injury in ischemia-reperfusion model 54. The underlying mechanisms of the contradiction remain to be studied. It has been reported that post-conditioning (PC) inhibits apoptosis induced by I/R, however, its myocardial protection is lost in the elderly heart 55, 56. Sun et al. reported that H_2_S restored the cardioprotective effect of PC and reduced I/R-induced ERS, the similar effects were obtained by using 4-PBA, which indicated that exogenous H_2_S restores PC-induced cardioprotection by inhibition of ERS in the aged cardiomyocytes 57 (Table 1). Although there are many studies about the protective effect of H_2_S on myocardium by influencing ER stress, the exact mechanism is not fully understood. Further researches are needed to provide a new way for the treatment of myocardial injury.

## 3. H_2_S influences endoplasmic reticulum stress in neurological diseases

In recent years, there have been many reports that H_2_S regulates ERS to inhibit neurological diseases. Homocysteine (Hcy), produced by demethylation of methionine 58, can induce ERS to lead the apoptosis of many types of neurons, including hippocampal and cortical neurons 59. The research by Li et al. demonstrated that intraventricular injection of Hcy impaired learning and memory function, reduced the production of endogenous H_2_S and increased the ERS of hippocampal cells, which suggested that Hcy-induced learning and memory loss was associated with reduced endogenous H_2_S production and increased hippocampal ERS 60. Similarly, the neurotoxicity to PC12 cells induced by arecoline is also involved with reduced endogenous H_2_S production and increased hippocampal ERS 61. Wei et al. reported that H_2_S downregulated Hcy-induced neuronal ERS and upregulated the expression of brain-derived neurotrophic factor (BDNF) in the hippocampus of rats. In addition, blocking BDNF-TrkB pathway by inhibitor could reverse the abovementioned effect of H_2_S. Overall, these findings suggested that H_2_S alleviated Hcy-induced neurotoxicity through reducing ERS by upregulating the BDNF-TrkB pathway 62. In PC12 cells, H_2_S also markedly inhibited homocysteine‑induced ERS and increased the protein level of silent mating type information regulation 2 homolog 1 (SIRT‑1) in the presence or absence of homocysteine treatment. Sirtinol, an inhibitor of SIRT‑1, eliminated the inhibitory effect of H_2_S on homocysteine‑induced ERS, which indicated that H_2_S protected PC12 cells against homocysteine‑induced ERS by upregulating SIRT‑1 63. The similar results were obtained in vivo 64 Increasing evidences suggest that diabetes can cause cognitive impairment and memory loss 65, 66. ERS-induced apoptosis in the hippocampus is the mechanism of diabetic cognitive impairment 67. Wei et al. reported that H_2_S improved cognitive impairment in diabetes mellitus by inhibiting ERS induced by hyperglycemia in hippocampus. Furthermore, the hippocampal endogenous H_2_S generation of diabetic rats was decreased, while this downregulation is reversed by exogenous H_2_S 68. These results suggested that the neuroprotective effect of H_2_S might be related to its promotion of endogenous H_2_S production in hippocampal cells. However, it has been reported that the endogenous H_2_S production in pancreas and liver of STZ-induced diabetic rats increased significantly 69. This conflict is probably due to that the concentration of endogenous H_2_S is diverse in different tissue. The mechanisms of H_2_S regulating ERS to protect nerve injury need to be further studied. ERS will be a new target of treatment for neurological diseases.

Depression is a chronic and recurrent serious mental disorder characterized by loss of happiness, emotional disorders and suicidal tendencies. It affects more than 10% of the world's population and causes a huge social burden 70, 71. It has been reported that rats exposed to chronic unpredictable mild stress (CUMS) exhibited many behavioral and neurobiological changes in depression 72. The research demonstrated that CUMS induced depression-like behavior, caused hippocampal ERS in rats and suppressed the production of endogenous H_2_S, while exogenous H_2_S alleviated the above depression-like behavior suggesting that H_2_S production disorder and ER stress in hippocampus played an important role in depressive behavior induced by CUMS 73. The further studies showed that exogenous H_2_S attenuated CUMS-induced depression-like behaviors by suppressing hippocampal ERS and increased the SIRT‑1 expression in rats. Moreover, the inhibition of SIRT‑1 by inhibitor reversed the protective effect of H_2_S and promoted CUMS-induced ERS. Collectively, these indicated that H_2_S inhibited CUMS-induced depressive-like behavior through suppressing CUMS-induced ERS by upregulating SIRT‑1 pathway 74. H_2_S also exerts its protection against the neurotoxicity of formaldehyde through overcoming ERS via upregulation of SIRT-1 75. In addition to the SIRT‑1 pathway, the BDNF/TrkB pathway is also related with the antidepressant effect of H_2_S. BDNF is an important endogenous neurotrophic factor, mainly expressed in hippocampus and cortex 76. H_2_S mitigates CUMS-induced depressive-like behaviors, induces the expressions of BDNF and p-TrkB proteins and inhibits ERS in the hippocampus of CUMS-induced rats 77. The inhibition of BDNF-TrkB pathway with K252a, an inhibitor of BDNF, reverses the protective role of H_2_S in CUMS-induced depressive-like behaviors and hippocampal ERS, which indicates that H_2_S plays an antidepressant-like effect through suppressing ERS via BDNF-TrkB pathway in CUMS-exposed rats 78 (Table 2). At present, the existing treatment of depression is often ineffective and cannot completely solve the symptoms. With the in-depth study of the mechanism of H_2_S antidepressant effect, the new H_2_S-related drugs will be provided for the treatment of depression. ERS will also become a new target for the treatment of depression.

## 4. H_2_S influences endoplasmic reticulum stress in respiratory diseases

Chronic obstructive pulmonary disease (COPD) can be defined as a disease characterized by exposure to harmful substances, leading to irreversible airflow restriction and shortness of breath 79, 80. Evidences suggest that ERS may play an important role in the development or pathology of COPD 81, 82. Cigarette smoke (CS) induces ERS and ERS-mediated apoptosis and suppresses the production of endogenous H_2_S to lead COPD, which is reversed by exogenous H_2_S 83. Intraperitoneal injection of endogenous H_2_S inhibitor in rat model of passive inhalation of CS aggravates these effects caused by CS; however, the ERS inhibitor suppresses CS-induced effects, which suggests that H_2_S may inhibit CS-induced bronchial epithelial cell apoptosis through suppressing ERS 84. Artery endothelial dysfunction induced by apoptosis of arterial endothelial cells is associated with the severity of COPD 85. Exogenous H_2_S reduces the apoptosis of pulmonary artery endothelial cells by suppressing ERS in a rat model of COPD 86. The specific signaling pathways involved in the above effect need to be further studied. The decrease of ERS and endogenous H_2_S are involved in the pathogenesis of acute lung injury (ALI). Exogenous H_2_S can play protectice role during the early stage of ALI by increasing ERS, which is contrary to the former statement 87. The reason needs to be studied (Table 3).

## 5. H_2_S influences endoplasmic reticulum stress in vascular disease

Vascular calcification (VC) refers to the abnormal deposition of calcium and phosphorus minerals on the wall of blood vessels; ERS-induced apoptosis plays a vital role in the development of VC 88, so inhibiting apoptosis is an effective treatment for VC. Yang et al. reported that H_2_S could inhibit VC and ERS of calcified aortic tissues. Furthermore, the ERS inducer Tm could block the ameliorated effect of H_2_S on VC, while the effect of the ER stress inhibitor PBA on VC in rats treated with vitamin D3 plus nicotine was similar as that of H_2_S.These indicated that H_2_S ameliorated VC by suppressing ERS. Moreover, the protein levels of phosphorylated AKT and Akt were both upregulated by H_2_S, suggesting that activation of the Akt signaling pathway is involved with the above effect of H_2_S 89. With the development of research, it will provide a new strategy and target for the prevention and treatment of VC. Endothelial dysfunction (ED) caused by inflammation is very important in the development of atherosclerosis (AS). Angiotensin II (AngII) is involved in the progression of ED, leading to atherosclerosis 90. There is evidence that ERS and ED are the key factors leading to AngII-induced cytotoxicity 91, 92. The results of Hu et al. revealed that AngII markedly induced cytotoxicity by promoting ERS and ED in human umbilical vein endothelial cells (HUVECs), which are reversed by H_2_S supplementation via inhibiting NF-кB signaling pathway 93 (Table 3). Similar results were obtained in other studies 94, 95. Whether ERS can directly induce ED and the molecular mechanism of interaction between ER stress and ED need to be studied. With the deepening of the research, it will certainly provide a new prevention and treatment of AS.

## 6. Conclusion

**Figure 3:**
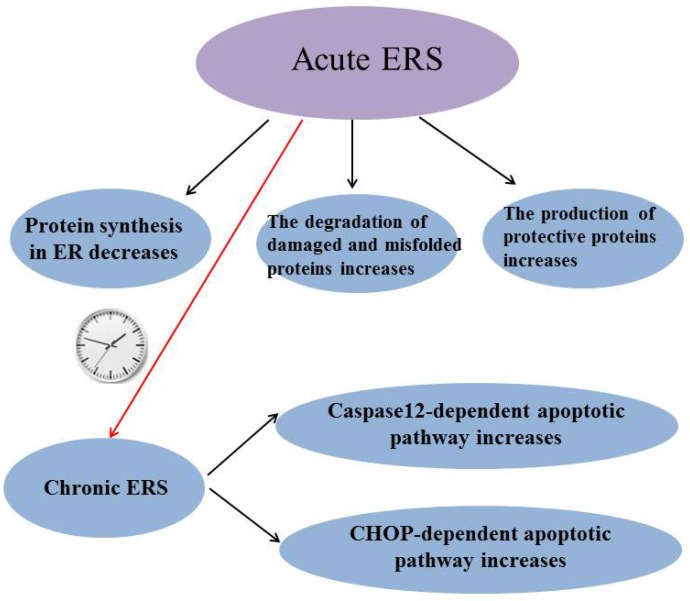
** Summary of the role of endoplasmic reticulum stress.** ERS: endoplasmic reticulum stress; CHOP:C/EBP-homologous protein.

ERS has been reported to be involved in many diseases and it is a “double-edged sword”: Acute ERS can reduce protein synthesis in ER, increase degradation of damaged and misfolded proteins and induce the production of protective proteins to alleviate stress-induced injury, while chronic ERS can induce caspase-12 dependent apoptotic pathway and C/EBP-homologous protein (CHOP) dependent apoptotic pathway to lead some diseases (Figure 3). So it is very important to study how to maintain ERS at an appropriate level. Although the mechanism of how prolonged ERS leads to disease is not fully understood, it is now clear that abnormal ERS can cause disease by inducing excessive reactive oxygen species (ROS).The suppression of excessive ERS will provide a way to prevent and treat many diseases. H_2_S has been shown to play a protective role in many diseases by inhibiting ERS, but individual research has reported that H_2_S inhibits diseases by promoting ERS; perhaps the reason is that the basic level of ERS varies in different tissues or different diseases have different effects on ERS. The mechanism of H_2_S regulating ERS in diseases needs further study. In conclusion, ERS may be a potential target for H_2_S therapy with the in-depth study of the effect of H_2_S on ERS.

## Figures and Tables

**Table 1:** H_2_S playes cardioprotective role by influencing endoplasmic reticulum stress (ERS)

Experimental models	Effects	Proposed mechanisms	References
Streptozotocin(STZ)-induced diabetic rats	Intraperitoneally administation of NaHS at 30 µmol/kg or 100 µmol/kg for 8 weeks could improve myocardial hypertrophy and myocardial collagen deposition in hearts of diabetic rats	Suppressing STZ‑induced ERS	35
AC16 cardiac cells treated with palmitic acid(PA)	Pretreatment AC16 cells with 100 μmol/L of NaHS could suppress the PA-induced myocardial injury	Suppressing PA‑induced ERS	45
Murine model of high fat diet (HFD)-induced cardiomyopathy	H_2_S therapy mitigated HFD-induced cardiac dysfunction	Suppressing cardiac ERS induced by HFD feeding	46
Model of hypoxia/reoxygenation in rat H9c2 cardiac myocytes.	H_2_S preconditioning significantly reduced myocardial infarct size, preserved left ventricular function, and inhibited I/R-induced cardiomyocyte apoptosis in vivo.	Attenuating I/R-induced ERS	49
Chronic intermittent hypoxia (CIH) model in rats	Inhibiting the production of endogenous H_2_S by PAG alleviated myocardial injury induced by CIH.	Reducing ERS induced by CIH	53
H_2_O_2_-induced H9C2 cells senescence model	Exogenous H_2_S restores PC-induced cardioprotection	Inhibition of ERS via down-regulating PERK-eIF 2α-ATF4, IRE 1α-XBP-1 and ATF 6 pathways	57

PAG:DL-propargylglycine; I/R:ischemia reperfusion; PC:post-conditioning; PERK:pancreatic endoplasmic reticulum kinase; IRE1α:inositol-requiring enzyme1α; ATF6(4): activating transcription factor 6(4); XBP-1:X-box binding protein 1; eIF2α:Eukaryotic initiation factor 2α.

**Table 2:** H_2_S influences endoplasmic reticulum stress (ERS) in neurological diseases

Experimental models	Effects	Proposed mechanisms	References
Adult male Sprague-Dawley rats were intracerebroventricularly injected with Hcy	H_2_S alleviated Hcy-induced neurotoxicity	Inhibiting ERS by upregulating the BDNF -TrkB pathway	62
Homocysteine-treated PC12 cells	Exogenous H_2_S significantly attenuated the homocysteine‑induced ERS response in hippocampal.	Inhibiting homocysteine‑induced ERS by upregulating SIRT‑1	63
Streptozotocin-induced diabetic rats	H_2_S improved cognitive impairment in diabetes mellitus	Suppressing hippocampal endoplasmic reticulum stress induced by hyperglycemia	68
Rat model of chronic unpredictable mild stress	H_2_S inhibited CUMS-induced depressive-like behavior.	Suppressing CUMS-induced ERS by upregulating SIRT‑1 pathway	74
Formaldehyde (FA)-induced PC12 cells	H_2_S exerts its protection against the neurotoxicity of FA.	Through overcoming ERS via upregulating SIRT‑1 pathway	75
Rat model of chronic unpredictable mild stress	H_2_S inhibited CUMS-induced depressive-like behavior.	Suppressing ERS via BDNF-TrkB pathway.	77,78

BDNF: brain-derived neurotrophic factor; TrkB: tyrosine protein kinase B; SIRT-1: silent mating type information regulation 2 homolog 1; CUMS: chronic unpredictable mild stress.

**Table 3:** H_2_S influences endoplasmic reticulum stress (ERS) in respiratory diseases and vascular diseases

Experimental models	Effects	Proposed mechanisms	References
Sprague-Dawley rats exposed to cigarette smoke (CS) generated from 20 commercial unfiltered cigarettes for 4 h/day, 7 days/week for 4 months	NaHS significantly inhibited CS-induced bronchial epithelial cell apoptosis in rat lungs	Inhibiting ERS	84
Rat model of chronic obstructive pulmonary disease established by means of passive smoke exposure and intratracheal injection with lipopolysaccharide (LPS)	Exogenous H_2_S reduced the apoptosis of pulmonary artery endothelial cells	Suppressing ERS	86
Rats with acute lung injury (ALI) induced by oleic acid (OA)	H_2_S could promote alveolar epithelial cell endoplasmic reticulum stress in rats with ALI.		87
Vitamin D3 plus nicotine (VDN) model of rats	H_2_S alleviated vascular calcification (VC) and phenotype transformation of vascular smooth muscle cells.	Inhibiting ERS via activation of the Akt signaling pathway	89
10-6 M AngII treated human umbilical vein endothelial cells (HUVECs)	H_2_S protected human umbilical vein endothelial cells (HUVECs) against AngII‑stimulated ET‑1 generation and subsequent cytotoxicity‑induced endoplasmic reticulum stress	Via inhibiting NF-кB signaling pathway	93

Akt:serine threonine kinase; NF-кB:nuclear factor kappa-B
